# Cyclical fluctuations of iron biomarkers in women: Diagnostic implications for iron deficiency

**DOI:** 10.1016/j.plabm.2025.e00512

**Published:** 2025-11-22

**Authors:** Sixtus Aguree, Arthur Owora, Patricia Silveyra

**Affiliations:** a Department of Applied Health Science, Indiana University School of Public Health-Bloomington, Bloomington, IN, USA; b Department of Pediatrics, School of Medicine, Indiana University, Indianapolis, IN, USA; c Department of Epidemiology and Biostatistics, School of Public Health, Indiana University, Bloomington, IN, USA; d Department of Environmental and Occupational Health, Indiana University Bloomington, Bloomington, IN, USA; e Department of Medicine, School of Medicine, Indiana University, Indianapolis, IN, USA

**Keywords:** Menstrual cycle, Iron biomarkers, Iron deficiency, Iron deficiency anemia, Serum ferritin, Transferrin saturation

## Abstract

**Background:**

Iron deficiency (ID) and iron deficiency anemia (IDA) are common in women of reproductive age, but the influence of menstrual cycle phase on iron biomarkers is not well defined and is often overlooked in clinical and public health assessments.

**Aim:**

To assess phase-specific variation in iron biomarkers and the prevalence of ID and IDA in non-pregnant women aged 18–44 years using 2003–2006 NHANES data.

**Methods:**

We analyzed 1484 women with complete reproductive and iron status data. Menstrual cycle phase was categorized as menstruation (day 1–5), follicular phase (6−15), early/mid luteal phase (16–23), and late luteal phase (24–35). Eight biomarkers were analyzed: serum iron (SI), transferrin saturation (%TS), soluble transferrin receptor (sTfR), ferritin, erythrocyte protoporphyrin (EPP), hemoglobin (Hb), mean corpuscular volume (MCV) and body iron index (BII). ID and IDA were defined using ferritin-, MCV- and BII-based diagnostic models. All statistical models accounted for the complex design of the NHANES survey.

**Results:**

SI and %TS were lowest during menstruation and increased across the cycle, peaking in the early/mid-luteal phase (SI: p = 0.001; %TS: p = 0.003). sTfR was highest during menstruation (p < 0.05) compared to other phases, consistent with increased iron requirements. Ferritin, EPP, Hb and MCV remained stable across phases. The prevalence of ID varied by model (10.5 %–22.0 %) but showed no consistent phase differences. In contrast, the prevalence of IDA decreased after menstruation, with composite IDA estimates dropping from 7.5 % during menstruation to 3.7 % in the late luteal phase (p = 0.033).

**Conclusions:**

Iron biomarkers and IDA prevalence vary systematically across the menstrual cycle, with iron status being lowest during menstruation and recovering in the luteal phase. Consideration of menstrual phase may improve diagnostic accuracy and interpretation of iron biomarkers in women of reproductive age.

## Highlights

• Iron biomarkers (serum iron, transferrin saturation, sTfR) fluctuate significantly across menstrual phases.
• Iron deficiency is most frequently detected in the early follicular phase.
• Considering menstrual phase may improve diagnostic accuracy for iron deficiency and anemia in reproductive-age women.

## 1 Introduction

The menstrual cycle plays a crucial role in women's health and has a significant impact on nutritional status, especially iron levels. Iron is essential for numerous biological functions, including oxygen transport, energy production [1]. Despite its importance, iron deficiency remains one of the most common micronutrient deficiencies worldwide, particularly in women of childbearing age [2]. In the United States, ID affects approximately one in ten women aged 15–49 years [3], and menstruation contributes significantly to this burden [4]. In the United States, recent NCHS(of the CDC) reports show that anemia is prevalent in women aged 15–49 years and has increased over time, underscoring the clinical burden of iron-related disorders in this age group [5]. Despite this, the impact of the menstrual cycle on micronutrient status, particularly iron biomarkers, is still not fully understood [6].

While the relationship between menstrual blood loss and ID is well known [7], the dynamic variations in iron biomarkers during the menstrual cycle have not been thoroughly investigated, especially for modern markers, such as soluble transferrin receptor (sTfR) and body iron index (BII). Several studies, including cross-sectional analyses and repeated measures studies, have shown that cyclical fluctuations in ferritin, transferrin saturation (T), and serum iron levels across the menstrual cycle can be significant. These fluctuations are most pronounced in the early follicular phase, and can lead to false-positive diagnoses of ID if the menstrual cycle phase is not considered [[8], [9], [10], [11]].

Previous studies have often been limited by small samples (typically a few dozen to ∼90 participants) or select populations (e.g., endurance athletes) [9,12,13], with the exception of that of the Second National Health and Nutrition Examination Survey (NHANES II, 1976–1980, n = 1712 women aged 18–44 years) [10,14]. Moreover, few studies have systematically examined cycle phase patterns for newer indices such as soluble transferrin receptor (sTfR) or body iron index (BII) [9,15]. The present study advanced our understanding of the menstrual cycle and iron biology, by including these new biomarkers in addition to well-established markers using data from the 2003–2006 cycles of NHANES IV. The 2003–2006 NHANES cycles were the only time when both menstrual cycle length and multiple iron biomarkers were simultaneously available.

By integrating responses to the Reproductive Health Questionnaire with laboratory measurements of iron status, this study aims to characterize menstrual cycle-related variations in iron biomarkers and assess how phase-specific differences influence the prevalence of ID and IDA in U.S. women aged 18–44 years.

## 2 Methods

### 2.1 Study population

We analyzed data from the 2003–2006 National Health and Nutrition Examination Survey (NHANES), a nationally representative cross-sectional survey of the civilian, noninstitutionalized population in the United States. NHANES uses a multistage probability design conducted by the U.S. Centers for Disease Control and Prevention (CDC) to assess the health and nutritional status of the U.S. population. The survey included a home interview, medical examination at a mobile examination center (MECs), and follow-up phone call. Standardized physical examinations, and laboratory tests conducted are performed during visits to the MECs. For this analysis, we restricted the sample to non-pregnant, menstruating women aged 18–44 years with complete biomarker and menstrual cycle information. After applying 4-year MEC screening weights, this subpopulation represents an estimated 33 million women in the United States.

### 2.2 Exposure: menstrual cycle phase

The menstrual phase was classified according to the self-reported cycle days and divided into four biologically meaningful groups: Menstruation (days 1–5), follicular phase (6−15), early/mid luteal phase (16–23) and late luteal phase (24–35) [16]. In the primary analyzes, menstruation was defined as the reference category. To allow estimation of all pairwise contrasts, the models were repeated with alternative reference groups (menstruation, follicular phase, late luteal phase).

### 2.3 Laboratory methods

Biomarkers were measured according to standardized NHANES protocols. All procedures adhered to NHANES quality assurance and control standards consistent with the 1988 Clinical Laboratory Improvement Amendments (CLIA), including calibration verification, control materials, and external proficiency testing. Detailed assay protocols have been published previously [[17], [18], [19]].

In brief, blood specimens were collected at NHANES MECs, where complete blood count (CBC), including mean corpuscular volume (MCV), was performed immediately on whole blood samples using the Beckman Coulter MAXM analyzer (Beckman Coulter, Fullerton, CA). Serum and erythrocyte aliquots were separated, frozen—typically at −20 °C or −70 °C depending on the analyte—and shipped to designated contract laboratories for biochemical analyses. Serum iron (SI) and total iron binding capacity (TIBC) were analyzed in frozen serum samples using a colorimetric assay on the LX20 analyzer (Beckman Coulter, Fullerton, CA). In this method, iron is released from transferrin using acetic acid, reduced to the ferrous state with hydroxylamine and thioglycolate, complexed with a chromogen (FerroZine) and quantified by absorbance at 560 nm. The TIBC was determined using the unsaturated iron binding capacity (UIBC) method, and the transferrin saturation (TS) was calculated as follows: TS (%) = (SI/TIBC) × 100.

Serum ferritin was measured by immunoturbidimetry on the 912 analyzer (Roche Diagnostics, Indianapolis, IN) using harmonization procedures to account for differences between assays in different collection cycles (BioRad IRMA in 2003 versus Roche/Hitachi in 2004–2006; BioRad Laboratories, Hercules, CA). To ensure consistency across survey years, NHANES applied piecewise linear regression equations to harmonize Bio-Rad IRMA values with Roche/Hitachi measurements. Soluble transferrin receptor (sTfR) concentrations were measured immunoturbidimetrically using Roche kits on the same analyzer (Roche Diagnostics, Mannheim, Germany). Erythrocyte protoporphyrin (EPP) was quantified from whole blood at the Wadsworth Center, New York State Department of Health, Trace Metals Laboratory (Albany, NY), using the NCCLS C42-A consensus method based on Sassa and Chisolm. Porphyrins and heme were extracted with a 4:1 ethyl acetate–acetic acid mixture, back-extracted into 1.5 M hydrochloric acid, and quantified fluorometrically against protoporphyrin IX standards verified by absorbance using Beer's Law.

### 2.4 Outcomes

Iron status was assessed using eight laboratory-based indicators covering multiple domains of iron metabolism and erythropoiesis: serum iron (SI; μg/dL), transferrin saturation (TS; %), mean corpuscular volume (MCV; fL), hemoglobin (Hb; g/dL), log-transformed serum ferritin (SF; μg/L), erythrocyte protoporphyrin (EPP; μmol/L RBC), log-transformed soluble transferrin receptor (sTfR; mg/L) and the body iron index (BII; mg/kg). The BII was calculated using the Cook et al. (2003) equation, which integrates the ferritin and sTfR concentrations to obtain a continuous scale ranging from negative (iron deficiency) to positive (iron sufficiency) [20].

In parallel, binary outcomes were derived from established thresholds for deficiency and anemia. Indicators included low ferritin (<15 μg/L) [21], low SI(60 μg/dL) [22], low TS (<20 %) [23], elevated EPP (>1.24 μmol/L RBC) [19,24], elevated sTfR (4.4 mg/L) [25], low Hb (<12.0 g/dL) [24], microcytosis (MCV <80 fL) [26] and low BII (<0 mg/kg) [20]. Three complementary models of ID were applied: the ferritin model (≥2 abnormal values between ferritin, transferrin saturation and erythrocyte protoporphyrin) [24], the MCV model (≥2 abnormal values between mean corpuscular volume, transferrin saturation and erythrocyte protoporphyrin) [18,27] and the body iron model (body iron index <0 mg/kg) [20]. To estimate BII, Roche sTfR concentrations were converted to values equivalent to the Flowers assay as recommended in prior comparative studies of the two assays [28,29]. Women who met the criteria of one of the models were classified as iron deficient. Iron deficiency anemia was defined as hemoglobin <12.0 g/dL in combination with each diagnostic model, yielding parallel indicators of IDA by ferritin, MCV, body iron, and a composite of “any IDA.

### 2.5 Covariates

The adjusted models included covariates that were selected a priori as established determinants of iron status: Age (continuous), ethnicity (non-Hispanic white, non-Hispanic black, Mexican American, other), body mass index (continuous), and smoking status (never, former, current).

### 2.6 Statistical analysis

All analyses incorporated the complex, multistage sampling design of the NHANES, which includes strata (SDMVSTRA), primary sampling units (SDMVPSU), and 4-year weights (WTMEC4YR) for mobile examination centers (MEC) to obtain nationally representative estimates. Variance estimation was performed using Taylor series linearization. Descriptive statistics included survey-weighted means and 95 % confidence intervals (CIs) for continuous biomarkers across menstrual phases. For skewed variables (ferritin and soluble transferrin receptor [sTfR]), means were calculated on the log scale. For categorical outcomes, survey-weighted means were interpreted as phase-specific prevalences of iron deficiency (ID) and iron deficiency anemia (IDA).

To formally test for differences between menstrual phases, survey-weighted linear regression was used for continuous biomarkers and survey-weighted logistic regression was used for binary outcomes. Each model was run in two specifications: (1) unadjusted (menstrual phase only) and (2) adjusted for age, race/ethnicity, body mass index (BMI) and smoking status. To allow for all pairwise comparisons, the regressions were repeated with rotated reference categories (menstruation, follicular phase, late luteal phase). Regression coefficients (β), odds ratios (ORs), 95 % CIs and design-adjusted p-values were reported.

Overall differences between phases were assessed using design-based Wald F-tests for continuous outcomes and design-adjusted Pearson F-tests for categorical outcomes. All tests were two-sided with α = 0.05, and no adjustments were made for multiple comparisons.

Analyses were performed using Stata/SE 19 (StataCorp, College Station, TX). NHANES protocols were approved by the National Center for Health Statistics Research Ethics Review Board, and all data are publicly available and de-identified.

## 3 Results

### 3.1 Study population and sample characteristics

The sample analyzed included 1484 nonpregnant, menstruating women aged 18–44 years from the 2003–2006 NHANES survey, representing approximately 33.9 million U.S. women after applying survey weights. The mean age was 31.7 years (SD = 6.2) and the mean BMI was 27.2 kg/m^2^ (SD = 5.6). Blood samples were collected throughout the menstrual cycle, with 19.3 % during menstruation (days 1–5), 32.7 % during the follicular phase (days 6–15), 25.9 % during the early/mid luteal phase (days 16–23) and 22.2 % during the late luteal phase (days 24–35). Approximately two-thirds of participants were white, 12 % were black, 15 % were of Mexican descent, and 7 % were of another ethnicity.

All analyzes considered the complex NHANES survey design. Adjusted models additionally considered age, race/ethnicity, BMI, and smoking status.

### 3.2 Iron biomarkers across the menstrual cycle

Several biomarkers of iron status fluctuated during the menstrual cycle. Serum iron concentrations were lowest during menstruation (72.8 μg/dL) and increased significantly during the follicular phase (β = +10.4 μg/dL, 95 % CI: 5.0–15.9, p = 0.001) and early/mid luteal phase (β = +14.9 μg/dL, 95 % CI: 6.3–23.6, p = 0.001) (Table 1; appendix 1). In the late luteal phase, the values decreased slightly (83.1 μg/dL; β = +10.3 μg/dL, 95 % CI: 1.5–19.1, p = 0.023). Transferrin saturation followed a similar pattern, increasing from 20.8 % during menstruation to 23.5 % in the follicular phase (β = +2.7 %, p = 0.002) and peaking at 24.6 % in the early/mid-luteal phase (β = +3.8 %, p = 0.003), with a borderline increase in the late luteal phase (23.2 %; β = +2.4 %, p = 0.052). In contrast, soluble transferrin receptor (natural log-transformed) was highest during menstruation (1.29 mg/L) and significantly lower in subsequent phases: follicular (β = −0.083, p = 0.004), early/mid luteal (β = −0.077, p = 0.008) and late luteal (β = −0.060, p = 0.031). This pattern suggests a transient increased iron requirement or decreased availability during menstruation.

**Table 1:** Mean serum ferritin, serum iron, serum transferrin saturation, whole blood erythrocyte protoporphyrin, mean cell volume, body iron index, and hemoglobin of non-pregnant females aged 18–44 y: NHANES 2003–2006 a^,^^b^.

Biomarkers	EFP	LFP	ELP	LLP
N (unweighted)	286	485	384	329
Serum Iron (μg/dL)	72.8 (67.2–78.5)^a^	83.3 (79.4–87.1)^b^	87.8 (81.9–93.6)^b^	83.1 (77.2–89.1)^b^
TS (%)	20.8 (19.1–22.5)^a^	23.5 (22.2–24.8)^b^	24.6 (23.1–26.1)^b^	23.2 (21.7–24.8)^ab^
MCV (fL)	89.0 (88.3–89.7)	88.7 (88.1–89.3)	89.4 (88.9–90.0)	88.8 (88.0–89.5)
Hb (g/dL)	13.5 (13.3–13.7)	13.5 (13.4–13.6)	13.7 (13.5–13.8)	13.6 (13.4–13.8)
SF (μg/L), c	3.53 (3.40–3.65)	3.54 (3.42–3.65)	3.56 (3.46–3.66)	3.64 (3.53–3.75)
EPP (μmol/L RBC)	0.98 (0.92–1.03)	1.00 (0.96–1.04)	0.97 (0.92–1.02)	0.97 (0.91–1.03)
sTfR (mg/L), c	1.29 (1.25–1.34)^a^	1.21 (1.17–1.25)^b^	1.22 (1.18–1.25)^b^	1.23 (1.19–1.28)^b^
BII (mg/kg)	4.85 (4.28–5.42)	5.21 (4.70–5.72)	5.26 (4.84–5.69)	5.51 (5.01–6.01)

EFP, early follicular phase (days 1–5: “menstruation”); LFP, late follicular/ovulation phase (days 6–15: “follicular phase”); ELP, early/mid-luteal phase ((days 16–23); LLP, late luteal phase (days 24–35). NHANES, National Health and Nutrition Examination Survey; SF, serum ferritin; TIBC, Total iron-binding capacity; TS, Transferrin saturation; EPP, erythrocyte protoporphyrin; MCV, Mean cell volume; Hb, Hemoglobin; sTfR, soluble transferrin receptor.

a Values are weighted arithmetic mean (95 % CI) unless otherwise indicated. Superscripts ^(a,b)^ mark survey-adjusted pairwise differences (two-tailed test, p < 0.05) from Wald test; cells sharing a letter do not differ.

b Calculated from SVY: mean command. Values estimates by menstrual cycle phase arithmetic means (95 % CI).

c Values are in natural log-transformed weighted means (95 % CI).

Other biomarkers including log ferritin (3.53–3.64 ng/ml across all phases), erythrocyte protoporphyrin (EPP), hemoglobin (Hb) and mean corpuscular volume (MCV) did not change significantly (all p > 0.20). Body iron index (BII) tended to increase from 4.85 mg/kg during menstruation to 5.51 mg/kg in the late luteal phase, although this change did not reach significance (p = 0.076).

Adjusted models confirmed these patterns. Serum iron remained significantly elevated in the follicular phase (β = +8.3 μg/dL, p = 0.016), early/mid luteal phase (β = +14.8 μg/dL, p = 0.002) and late luteal phase (β = +9.3 μg/dL, p = 0.028) compared to menstruation. Transferrin saturation was also higher in the follicular phase (β = +2.1 %, p = 0.037) and in the early/mid luteal phase (β = +3.8 %, p = 0.004), with a borderline increase in the late luteal phase (p = 0.063). Hemoglobin was slightly but significantly increased in the early/mid luteal phase (β = +0.21 g/dL, p = 0.021). sTfR remained lower in the follicular (β = −0.07, p = 0.031) and early/mid luteal phase (β = −0.07, p = 0.016), with a borderline decrease in the late luteal phase (p = 0.087).

### 3.3 Iron deficiency and anemia outcomes

Unadjusted logistic regression models showed no significant association between menstrual phase and isolated iron deficiency, regardless of whether this was defined by ferritin, MCV, body iron index or a composite criterion (all p > 0.20) (Fig. 1A, Fig. 1BA and B; Appendix 2). During menstruation, the prevalence of ID ranged from 12.9 % (BII model) to 24.3 % (ferritin model) which marginally higher than to 8.0–19.0 % in the late luteal phase. Across all phases, ID was consistently lowest in the body iron model (10.5 % overall) and highest in the ferritin model (21.4 % overall). While isolated ID did not vary significantly between phases (all p > 0.20), iron deficiency anemia (IDA) showed clear phase-specific patterns. In unadjusted analyzes, the prevalence of IDA, defined by ferritin model, decreased from 6.9 % during menstruation to 3.5 % in the late luteal phase (p = 0.071). A similar decrease was observed in IDA defined by BII (from 5.2 % to 2.3 %, p = 0.084), while IDA defined by MCV model peaked in the follicular phase (7.9 %) and was lowest in the early/mid-luteal phase (∼3 %; p = 0.022). A composite definition showed that IDA prevalence decreased from 7.5 % during menstruation to 3.7 % in the late luteal phase (p = 0.033).

**Fig. 1A:**
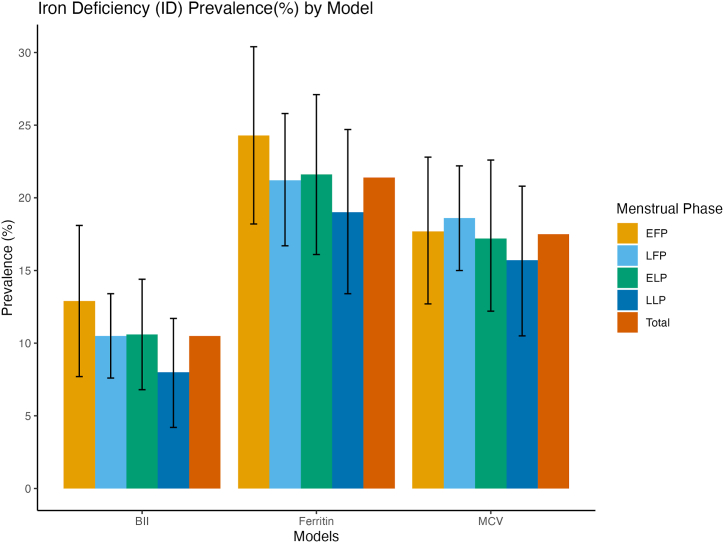
Prevalence (%) of ID by menstrual phase among non-pregnant females aged 18–44 y: NHANES 2003-2006 EF P, early follicular phase (days 1–5: “menstruation”); LFP, late follicular/ovulation phase (days 6–15: “follicular phase”); ELP, early/mid-luteal phase ((days 16–23); LLP, late luteal phase (days 24–35). NHANES, National Health and Nutrition Examination Survey.

**Fig. 1B:**
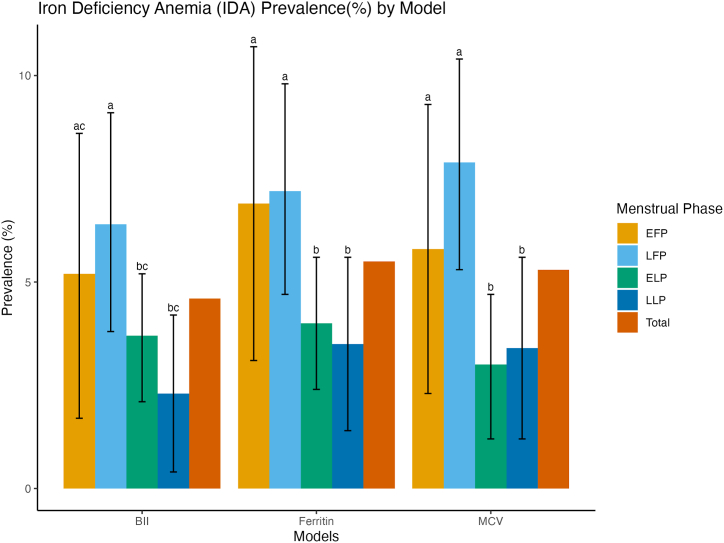
Prevalence (%) of IDA by menstrual phase among non-pregnant females aged 18–44 y: NHANES 2003-2006 EF P, early follicular phase (days 1–5: “menstruation”); LFP, late follicular/ovulation phase (days 6–15: “follicular phase”); ELP, early/mid-luteal phase ((days 16–23); LLP, late luteal phase (days 24–35). NHANES, National Health and Nutrition Examination Survey. ^a,b,c^ Within the same analytical model (e.g., BII model), values assigned different superscript letters (a, b, c) denote statistically significant differences in prevalence estimates (p < 0.05, two-tailed *t*-test). Differences in prevalence across menstrual phases were evaluated using survey-adjusted logistic regression models. The Wald test was used to assess the significance of model coefficients, and p-values were derived from the survey-weighted estimation. A two-sided p-value less than 0.05 was considered statistically significant.

Regression analyzes confirmed these trends. Compared to menstruation, the early/mid luteal phase was associated with a significantly lower likelihood of IDA across all models: Ferritin (OR = 0.53, p = 0.022), MCV (OR = 0.36, p = 0.013), BII (OR = 0.55, p = 0.045), and Composite (OR = 0.48, p = 0.008). The late luteal phase also offered protection: ferritin (OR = 0.46, p = 0.027), MCV (OR = 0.41, p = 0.021), BII (OR = 0.34, p = 0.018) and composite (OR = 0.44, p = 0.018). After adjusting for covariates, these associations persisted and often strengthened. For example, the adjusted probability of composite IDA was reduced by 69 % in the early/mid-luteal phase (OR = 0.31, 95 % CI: 0.17–0.58, p < 0.001) and by 56 % in the late luteal phase (OR = 0.44, 95 % CI: 0.21–0.91, p = 0.029). In contrast, the follicular phase (days 6–15) did not differ significantly from menses in IDA risk (all p > 0.20).

Overall, iron status fluctuated systematically during the menstrual cycle. Serum iron and transferrin saturation increased after menstruation and peaked in the early/mid-luteal phase, while sTfR levels were transiently elevated only during menstruation. These biomarker shifts resulted in a lower likelihood of iron deficiency anemia outside of menstruation, particularly in the early/mid and late luteal phases, where the protective associations persisted after multivariate adjustment. However, isolated iron deficiency showed no consistent differences between phases. Taken together, these results emphasize the importance of menstrual timing in the assessment of iron biomarkers in women of reproductive age and suggest that the premenstrual period represents a window of increased susceptibility to functional iron deficiency.

## 4 Discussion

In this nationally representative cohort of US women of reproductive age, iron status varied systematically across the menstrual cycle. The early follicular phase, corresponding to menstruation, was characterized by the lowest serum iron, transferrin saturation (%TS), and body iron index (BII), along with the highest prevalence of iron deficiency (ID) and iron deficiency anemia (IDA). In contrast, iron biomarkers progressively improved in the early/mid and late luteal phase, with serum iron and BII reaching a peak and the prevalence of ID/IDA reaching its lowest level. These results highlight the dynamic regulation of iron homeostasis during the menstrual cycle and emphasize the importance of considering the timing of menstruation when assessing iron status [9,11].

The nadir of serum iron and %TS during menstruation is consistent with physiologic iron loss through menstrual blood loss (MBL), which has been shown to contribute to iron deficiency in women [7,8,30]. Elevated soluble transferrin receptor (sTfR) concentrations during menstruation also indicate a transient increase in iron requirements or decreased availability, which is consistent with previous biomonitoring studies and BII data [15,31]. These findings are consistent with smaller clinical and athletic studies reporting low iron availability and hepcidin suppression during menstruation, followed by recovery later in the cycle [9,32]. Compared to previous research, which often relied on small clinical cohorts (21–90 participants), our study relies on a large, nationally representative sample. Previous work has shown that iron levels rebound after menstruation and that there is a strong association between MBL and ferritin levels [[7], [8], [9],^33^]. Our analysis extends this literature by capturing cycle-specific variation in several biomarkers, including sTfR and composite indices, in a population-based survey in the United States.

These findings have important clinical implications. Standard diagnostic thresholds for ferritin, %TS or hemoglobin may produce different results depending on when blood is drawn during the menstrual cycle, which may lead to misclassification. This suggests that incorporating the menstrual phase into clinical protocols could improve diagnostic accuracy.

Some limitations should be noted. The menstrual phase was based on self-report, which introduces potential recall bias, and the NHANES study does not include direct measurements of MBL. Hepcidin, an important regulator of iron metabolism, was also not measured, limiting insight into mechanistic pathways such as estrogen-mediated suppression or erythropoiesis-driven regulation after menstruation [34,35]. Nonetheless, the large sample size, nationally representative design, standardized assays, and comprehensive biomarker panel strengthen confidence in these results.

In conclusion, our analysis shows that the phase of the menstrual cycle significantly influences iron biomarkers, particularly serum iron, %TS, and sTfR concentrations. These variations could affect how iron status is assessed, indicating that reference ranges or diagnostic standards for ID and IDA should account for menstrual cycle phase in women of reproductive age. Future research should expand this, including a larger sample size, to help in developing phase-specific diagnostic thresholds and algorithms that integrate multiple markers to better account for physiologic variation.

## CRediT authorship contribution statement

**Sixtus Aguree:** Writing – original draft, Software, Methodology, Investigation, Formal analysis, Data curation, Conceptualization. **Arthur Owora:** Writing – review & editing, Methodology, Investigation, Formal analysis. **Patricia Silveyra:** Writing – review & editing, Methodology, Investigation.

## Author declarations

The authors declare that they have no known competing financial interests or personal relationships that could have appeared to influence the work reported in this paper.

## Ethics

Institutional review board approval was not required for this study as the NHANES survey data is publicly accessible, and the protocol was already approved. A description of the procedures for collecting the NHANES data can be found on the Centers for Disease Control and Prevention website and elsewhere (Available from http://www.cdc.gov/nchs/nhanes.htm (accessed October 25, 2023)^.^

## Funding disclosure

No external funding was received for this study.

## Declaration of competing interest

The authors declare that they have no known competing financial interests or personal relationships that could have appeared to influence the work reported in this paper.

## Figures and Tables

**Appendix Table 1:** Mean differences in iron biomarkers by menstrual cycle phase among non-pregnant females aged 18–44 y: NHANES 2003–2006 ^1^

	EFP	LFP	ELP	LLP
	Reference	β (95 % CI), p	β (95 % CI), p	β (95 % CI), p
Unadjusted model ^2^				
Serum iron (μg/dL)	1	10.42 (4.96–15.88), p = 0.001 ∗∗	14.94 (6.28–23.59), p = 0.001 ∗∗	10.31 (1.50–19.13), p = 0.023 ∗
%TS (%)	1	2.67 (1.09–4.26), p = 0.002 ∗∗	3.79 (1.43–6.16), p = 0.003 ∗∗	2.41 (−0.03–4.85), p = 0.052 †
MCV (fL)	1	−0.28 (−1.24–0.68), p = 0.555	0.44 (−0.46–1.34), p = 0.329	−0.24 (−1.31–0.83), p = 0.647
Hemoglobin (g/dL)	1	0.00 (−0.19–0.19), p = 0.987	0.17 (−0.03–0.37), p = 0.094 †	0.13 (−0.12–0.37), p = 0.298
log Ferritin	1	0.01 (−0.14–0.17), p = 0.882	0.03 (−0.15–0.22), p = 0.708	0.11 (−0.04–0.27), p = 0.150
EPP (μmol/L)	1	0.04 (−0.06–0.14), p = 0.446	−0.01 (−0.11–0.09), p = 0.816	0.01 (−0.14–0.15), p = 0.940
log sTfR	1	−0.08 (−0.14–0.03), p = 0.004 ∗∗	−0.08 (−0.13–0.02), p = 0.008 ∗∗	−0.06 (−0.11–0.01), p = 0.031 ∗
BII	1	0.36 (−0.36–1.08), p = 0.314	0.41 (−0.38–1.21), p = 0.297	0.66 (−0.07–1.39), p = 0.076 †
Adjusted model ^3^				
Serum iron (μg/dL)	1	8.25 (1.65–14.85), p = 0.016 ∗	14.81 (5.76–23.86), p = 0.002 ∗∗	9.34 (1.09–17.59), p = 0.028 ∗
%TS (%)	1	2.09 (0.13–4.05), p = 0.037 ∗	3.81 (1.34–6.29), p = 0.004 ∗∗	2.23 (−0.13–4.59), p = 0.063
MCV (fL)	1	−0.58 (−1.66–0.50), p = 0.283	0.47 (−0.54–1.48), p = 0.348	−0.22 (−1.29–0.85), p = 0.676
Hemoglobin (g/dL)	1	−0.06 (−0.27–0.15), p = 0.562	0.21 (0.03–0.39), p = 0.021 ∗	0.08 (−0.15–0.32), p = 0.470
log Ferritin	1	−0.02 (−0.19–0.15), p = 0.816	0.03 (−0.17–0.22), p = 0.780	0.10 (−0.06–0.27), p = 0.223
EPP (μmol/L)	1	0.07 (−0.04–0.17), p = 0.224	−0.02 (−0.13–0.08), p = 0.652	0.02 (−0.12–0.17), p = 0.749
log sTfR	1	−0.07 (−0.13–0.01), p = 0.031 ∗	−0.07 (−0.13–0.01), p = 0.016 ∗	−0.05 (−0.11–0.01), p = 0.087
BII	1	0.20 (−0.60–1.00), p = 0.612	0.38 (−0.46–1.22), p = 0.358	0.59 (−0.20–1.39), p = 0.136

EFP, early follicular phase (days 1–5: “menstruation”); LFP, late follicular/ovulation phase (days 6–15: “follicular phase”); ELP, early/mid-luteal phase ((days 16–23); LLP, late luteal phase (days 24–35). NHANES, National Health and Nutrition Examination Survey.

*Notes:* β = regression coefficient for difference vs. menstruation.

Models unadjusted, menstrual phase only. All analyses survey-weighted with strata, PSU, and 4-year MEC weights. † borderline (p ~ 0.05); ∗p < 0.05; ∗∗p < 0.01.

Models adjusted for age, race/ethnicity, BMI, and smoking status. All analyses survey-weighted with strata, PSU, and 4-year MEC weights.

**Appendix Table 2:** Odds ratios for iron deficiency and iron deficiency anemia by menstrual cycle phase among non-pregnant females aged 18–44 y: NHANES 2003–2006 ^1^

	EFP	LFP	ELP	LLP
	Reference	OR (95 % CI), p	OR (95 % CI), p	OR (95 % CI), p
Unadjusted model ^2^				
Anemia	1	0.97 (0.49–1.93), p = 0.938	0.55 (0.30–1.00), p = 0.050 †	0.53 (0.25–1.10), p = 0.088
BII model	1	0.80 (0.46–1.37), p = 0.404	0.81 (0.41–1.58), p = 0.516	0.59 (0.28–1.21), p = 0.142
Ferritin model	1	0.84 (0.58–1.22), p = 0.343	0.86 (0.54–1.36), p = 0.502	0.73 (0.42–1.27), p = 0.256
MCV model	1	1.06 (0.71–1.59), p = 0.766	0.97 (0.57–1.64), p = 0.898	0.86 (0.49–1.52), p = 0.594
Any model	1	0.82 (0.58–1.16), p = 0.244	0.78 (0.50–1.20), p = 0.244	0.73 (0.44–1.21), p = 0.212
BII model	1	1.26 (0.51–3.11), p = 0.598	0.70 (0.31–1.57), p = 0.374	0.43 (0.14–1.30), p = 0.128
Ferritin model	1	1.05 (0.50–2.22), p = 0.888	0.56 (0.27–1.16), p = 0.113	0.49 (0.20–1.17), p = 0.103
MCV model	1	1.38 (0.64–2.95), p = 0.395	0.49 (0.21–1.14), p = 0.095 †	0.57 (0.22–1.49), p = 0.240
Any model		1.07 (0.52–2.21), p = 0.843	0.52 (0.26–1.02), p = 0.057 †	0.47 (0.20–1.10), p = 0.080 †
Adjusted model ^3^	1			
Anemia	1	1.23 (0.56–2.71), p = 0.587	0.46 (0.25–0.84), p = 0.013 ∗	0.64 (0.29–1.40), p = 0.252
BII model	1	0.94 (0.48–1.83), p = 0.844	0.82 (0.38–1.76), p = 0.597	0.65 (0.29–1.48), p = 0.294
Ferritin model	1	0.94 (0.60–1.46), p = 0.765	0.82 (0.49–1.37), p = 0.439	0.76 (0.43–1.34), p = 0.338
MCV model	1	1.22 (0.79–1.88), p = 0.365	0.90 (0.49–1.67), p = 0.738	0.95 (0.53–1.69), p = 0.845
Any model	1	0.91 (0.61–1.37), p = 0.640	0.73 (0.45–1.19), p = 0.199	0.77 (0.45–1.31), p = 0.316
BII model	1	1.56 (0.58–4.20), p = 0.364	0.66 (0.30–1.45), p = 0.290	0.54 (0.17–1.73), p = 0.289
Ferritin model	1	1.29 (0.57–2.94), p = 0.533	0.49 (0.24–0.97), p = 0.041 ∗	0.59 (0.24–1.50), p = 0.259
MCV model	1	1.79 (0.76–4.20), p = 0.176	0.38 (0.15–0.99), p = 0.047 ∗	0.76 (0.26–2.16), p = 0.591
Any model		1.37 (0.61–3.11), p = 0.434	0.43 (0.22–0.84), p = 0.016 ∗	0.60 (0.25–1.46), p = 0.251

EFP, early follicular phase (days 1–5: “menstruation”); LFP, late follicular/ovulation phase (days 6–15: “follicular phase”); ELP, early/mid-luteal phase ((days 16–23); LLP, late luteal phase (days 24–35). NHANES, National Health and Nutrition Examination Survey.

*Notes:* OR <1 indicates lower odds compared with menstruation.

Models unadjusted, menstrual phase only. All analyses survey-weighted with strata, PSU, and 4-year MEC weights. † borderline (p ~ 0.05); ∗p < 0.05; ∗∗p < 0.01.

Models adjusted for age, race/ethnicity, BMI, and smoking status. All analyses survey-weighted with strata, PSU, and 4-year MEC weights.

## Data Availability

Data will be made available on request.
